# Cytoreductive Nephrectomy and Overall Survival of Patients with Metastatic Renal Cell Carcinoma Treated with Targeted Therapy—Data from the National Renis Registry

**DOI:** 10.3390/cancers12102911

**Published:** 2020-10-10

**Authors:** Alexandr Poprach, Milos Holanek, Renata Chloupkova, Radek Lakomy, Michal Stanik, Ondrej Fiala, Bohuslav Melichar, Katerina Kopeckova, Milada Zemanova, Igor Kiss, Igor Penka, Julia Bohosova, Tomas Buchler

**Affiliations:** 1 Department of Comprehensive Cancer Care, Masaryk Memorial Cancer Institute, 656 53 Brno, Czech Republic; holanek@mou.cz (M.H.); lakomy@mou.cz (R.L.); kiss@mou.cz (I.K.); 2 Department of Comprehensive Cancer Care, Faculty of Medicine, Masaryk University, 625 00 Brno, Czech Republic; 3 Institute of Biostatistics and Analyses, Faculty of Medicine, Masaryk University, 625 00 Brno, Czech Republic; chloupkova@iba.muni.cz; 4 Department of Urologic Oncology, Masaryk Memorial Cancer Institute and Masaryk University, 656 53 Brno, Czech Republic; stanik@mou.cz; 5 Department of Oncology, University Hospital, 323 00 Pilsen, Czech Republic; ondrej.fiala@fnplzen.cz; 6 Department of Oncology, Palacky University Medical School and Teaching Hospital, 779 00 Olomouc, Czech Republic; Bohuslav.melichar@upol.cz; 7 University Hospital in Hradec Kralove, 500 05 Hradec Kralove, Czech Republic; 8 Department of Oncology, Motol University Hospital and 2nd Faculty of Medicine Charles University, 150 06 Prague, Czech Republic; katerina.kopeckova@fnmotol.cz; 9 Department of Oncology, First Faculty of Medicine Charles University and General University Hospital, 120 00 Prague, Czech Republic; milada.zemanova@vfn.cz; 10 Department of Surgery, University Hospital Bohunice, Faculty of Medicine, Masaryk University, 625 00 Brno, Czech Republic; penka.igor@fnbrno.cz; 11 Central European Institute of Technology (CEITEC), University Campus Bohunice, Masaryk University, 625 00 Brno, Czech Republic; julia.bohosova@ceitec.muni.cz; 12 Department of Oncology, First Faculty of Medicine, Thomayer Hospital and Charles University, 140 59 Prague, Czech Republic; tomas.buchler@ftn.cz

**Keywords:** metastatic renal cell carcinoma, targeted therapy, cytoreductive nephrectomy, overall survival

## Abstract

**Simple Summary:**

The treatment of metastatic renal cell carcinoma is traditionally initiated with the removal of the diseased kidney with the tumor in many patients. However, there is ongoing controversy about the benefit of kidney removal if targeted therapy is used. The present paper analyses a large cohort of patients, and the results indicate that primary tumor removal should still be strongly considered in patients who are treated with targeted therapies.

**Abstract:**

The role of cytoreductive nephrectomy (CN) in treatment of locally advanced or metastatic renal cell carcinoma (mRCC) in the era of targeted therapies (TT) is still not clearly defined. The study population consisted of 730 patients with synchronous mRCC. The RenIS (Renal carcinoma Information System) registry was used as the data source. The CN/TT cohort included patients having CN within 3 months from the mRCC diagnosis and subsequently being treated with TT, while the TT cohort included patients receiving TT upfront. Median progression-free survival from the first intervention was 6.7 months in the TT arm and 9.3 months in the CN/TT patients (*p* < 0.001). Median overall survival was 14.2 and 27.2 months, respectively (*p* < 0.001). Liver metastasis, high-grade tumor, absence of CN, non-clear cell histology, and MSKCC (Memorial Sloan-Kettering Cancer Center) poor prognosis status were associated with adverse treatment outcomes. According to the results of this retrospective study, patients who underwent CN and subsequently were treated with TT had better outcomes compared to patients treated with upfront TT. The results of the study support the use of CN in the treatment algorithm for mRCC.

## 1. Introduction

Cytoreductive nephrectomy (CN) is one of the most controversial interventions in the treatment of metastatic renal cell carcinoma (mRCC). In the cytokine therapy era, CN was considered a mainstay of treatment for fit patients in cases where it led to substantial reduction of total tumor mass [1,2,3]. CN was even reported to result in spontaneous regression of metastases in rare cases [4,5]. 

The role of CN is less clear if targeted therapies (TT) are used as systemic agents for mRCC. CARMENA, the only prospective phase III trial studying CN in the context of TT, showed that the omission of CN and upfront initiation of sunitinib therapy in patients with synchronous mRCC is non-inferior compared to CN followed by sunitinib [6]. However, the CARMENA trial has been criticized and the conclusions have not been universally accepted [7], partly because of the contradiction with large retrospective studies [8,9,10,11] and meta-analyses [12,13,14]. The CARMENA study suffered from slow accrual that led to premature closure after the second interim analysis. Subsequently, the Steering Committee approved the results of the first interim analysis to be used as the overall trial results. In addition, as many as 44% of patients in the CN-sunitinib arm and 41.5% of patients in the sunitinib-only arm had poor risk features as defined by the Memorial Sloan Kettering Cancer Center (MSKCC) prognostic criteria [15], whereas the benefit of CN had only been postulated for intermediate-prognosis patients in previous reports [9,16,17]. Importantly, the majority of patients enrolled in CARMENA had a relatively high metastatic burden with the median of two metastatic sites. Furthermore, 17% of patients randomized to the sunitinib-only arm eventually underwent CN and, in the other study arm, in 7% of patients, CN was planned but not carried out. All these patients were included in the published analysis [6]. 

In the present retrospective study using data from the RenIS registry, we analyzed data of patients with de-novo mRCC treated with TT who underwent CN within three months of diagnosis and compared their progression-free survival (PFS) and OS (overall survival) with patients who did not have CN and were treated with TT only. In addition, intermediate-risk patients were analyzed per number of MSKCC risk factors. 

## 2. Results

### 2.1. Patient Population and Baseline Characteristics

There were 458 patients with synchronous metastatic disease and CN ≤ 3 months post-mRCC diagnosis followed by TT (CN/TT arm) and 272 patients without CN (TT arm). Median age at diagnosis was 64 and 62 years in the CN/TT and TT cohorts, respectively, and 95% of patients had clear cell renal cell carcinoma histology. Table 1 shows patient baseline characteristics. The site of metastasis is shown in Table S1.

### 2.2. Treatment Outcomes

While the overall response rate (ORR) was similar for the two analyzed cohorts, the disease control rate (DCR) favored the CN/TT patients. In the TT arm, overall response rate (ORR) was 19%, with complete response (CR) and partial response (PR) observed in 0.4% and 18.5% of patients, respectively. In the CN/TT, ORR was 24.4% CR (CR 2.9%, PR 21.4%). However, the difference in ORR was not statistically significant (*p* = 0.116). Stable disease was observed in 30.2% and 35.4% of patients in the TT and CN/TT arms, respectively. The disease control rate (DCR) was significantly higher in CN patients, reaching 49.2% and 59.8% for the TT and CN/TT arm, respectively (*p* = 0.011). 

**Figure 1:**
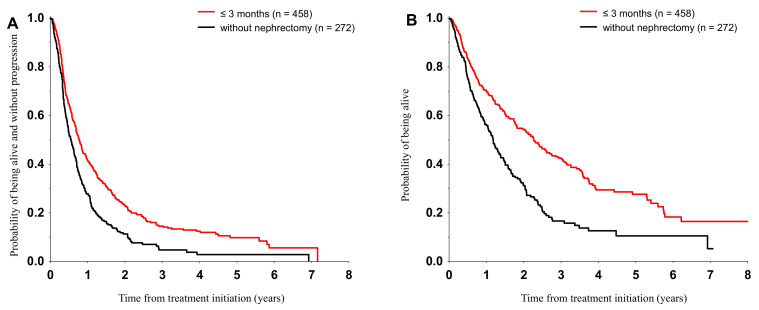
Progression-free (**A**) and overall survival (**B**) from first treatment strategy. All the differences were statistically significant at *p* < 0.001.

Median PFS was 6.7 months (95% CI: 5.5–7.8) in the TT arm and 9.3 months (95%: CI 8.3–10.4) in the CN/TT patients (*p* < 0.001). The median OS was 14.2 months (95% CI: 12.1–16.2) and 27.2 months (95% CI: 22.3–32.1), respectively (*p* < 0.001) (Figure 1).

### 2.3. Subgroup Analysis

The benefit of CN on PFS and OS was evident irrespective of age. Patients ≤65 years had a median PFS of 7.6 months (95% CI: 5.9–7.3) in the TT arm and 8.8 months (95% CI: 7.3–9.3) in the CN/TT arm. Median OS was 15 months (95% CI: 12.7–17.2) and 28.7 months (95% CI: 19.4–38.0) for the TT and CN/TT, respectively. In patients >65 years of age, median PFS was 5.8 months (95% CI: 4.3–7.3) and 10.1 months (95% CI: 8.0–12.2) and median OS was 12.5 months (8.3–16.6) and 26.3 months (95% CI: 20.0–32.5) for the TT and CN/TT cohorts, respectively. All the above differences were statistically significant at *p* < 0.001. Age at TT initiation had no statistically significant impact on PFS or OS in the multivariate analysis (Table 2).

Similarly, tumor grade was not a factor that significantly altered the benefit of nephrectomy. Patients with low-grade tumors had PFS of 8.1 months (95% CI: 6.7–9.5) versus 12 months (95% CI: 9.0–15.0) and an OS of 19.0 months (95% CI: 13.2–24.8) versus 31.0 months (95% CI: 22.1–39.9), while patients with high-grade tumors had PFS of 4.4 months (95% CI: 3.3–5.6) and 8.3 months (95% CI: 6.9–9.7), and an OS of 10.1 (95% CI: 7.6–12.7) and 23.6 months (95% CI: 17.8–29.4), for the TT and CN/TT cohorts, respectively. All the differences were statistically significant at *p* < 0.001).

There was a major difference in outcomes between intermediate-risk patients with one versus two risk factors, but both subgroups derived benefit from CN (Table 3). 

## 3. Discussion

The present analysis strongly suggests that CN is an independent, favorable predictive factor for treatment outcomes in patients with synchronous mRCC treated with TT. We conducted a subanalysis of the effect of CN on survival in the MSKCC intermediate-risk patients with one or two risk factors. The findings indicate that the benefit of CN is maintained in patients with adverse risk factors, regardless of age. 

The present data are in accordance with other retrospective studies. Heng et al. analyzed 1658 patients with synchronous mRCC treated with TT. Median OS was 9.5 months and 20.6 months in the TT-only and TT/CN patients, respectively. However, the results might have been affected by a more favorable prognostic profile in the CN/TT patients. The study also showed that patients with four or more International Metastatic Renal Cell Carcinoma Database Consortium (IMDC) prognostic factors did not benefit from CN [9]. Choueiri et al. showed in a similar retrospective study of 314 patients that CN patients had better OS than non-CN patients (19.8 vs. 9.4 months, respectively). However, again there were imbalances in patient characteristics: CN patients were younger and had better performance status, lower levels of corrected calcium, and a lower number of metastatic sites. Patients ≥75 years did not benefit from CN, in contrast with present findings suggesting similar benefits from CN in younger and older patients. Choueiri et al. also confirmed that poor-risk patients as defined by the MSKCC or the IMDC criteria did not benefit from CN [18]. The results of our study further support findings published by Zhao et al. that patients with both high- and low-grade tumors benefit from CN [19]. 

Important updates to the CARMENA study after a median follow-up of 61.5 months were presented at the American Society of Clinical Oncology 2019 Annual Meeting. The authors conducted a sub-analysis of IMDC intermediate prognosis patients, focusing on the difference in OS between patients with one versus two prognostic factors, and they explored whether the number of metastatic sites affected patient survival. In an update analysis of the CARMENA trial, patients with one prognostic factor benefited from CN, with the median OS of 31.4 months versus 25.2 months without CN [20], but patients with two IMDC risk factors did not, with median OS of 16.6 months and 31.2 months for patients with versus without CN, respectively. As the interval from RCC diagnosis to the initiation of treatment of less than year is a risk factor in both MSKCC and IMDC models, this is necessarily the single risk factor present in intermediate-risk patients and one risk factor. The first version of MSKCC criteria included previous nephrectomy instead of the treatment-free interval [21]. Thus, nephrectomy “converts” these patients into favorable-risk patients according the original MSKCC model. Patients with two risk factors remain intermediate-risk even after CN according to the older version of MSKCC classification. Therefore, this result from a prospective study seems to validate the older version of MSKCC if CN is considered. 

SURTIME was another prospective trial, a phase II study aiming to determine whether a period of sunitinib therapy before CN improves the outcome compared with immediate CN followed by the same agent. Although the study was underpowered and eventually closed due to poor accrual, the results indicated that deferred CN may be superior to early CN followed by sunitinib, with median OS of 32.4 months versus 15.1 months, respectively. In addition, a recent pooled analysis suggested that in intermediate-risk patients, deferred CN in patients not progressing on first-line vascular endothelial growth factor (VEGF)-directed therapy is superior to immediate CN followed by systemic treatment [22].

These results are in accordance with a post-hoc analysis of 40 patients in the sunitinib only arm of CARMENA, who, for different reasons, underwent nephrectomy. The OS of the CN patients was 48.5 months, while it reached 15.7 months in non-CN patients [20]. 

To summarize, the results of prospective studies suggest that CN still plays a role in patients with one IMDC risk factor and that patients not progressing on anti-VEGF therapy may benefit from later CN, although the question of upfront versus deferred CN remains unresolved and deserves a prospective trial. 

Several biological hypotheses have been put forward to explain the apparent benefit of cytoreductive nephrectomy in patients with metastatic disease. RCC is the prime model for spatial tumor heterogeneity. Gerlinger et al. clearly showed that most genetic driver mutations in RCC are subclonal, and only approximately one-third are present in all regions of the primary tumor and metastases [23,24]. Therefore, volume reduction may result in removal or limitation of tumor clones that may be resistant to the therapy and represent foci of future progressive disease. Immunological mechanisms may play a role in superior survival of patients with mRCC after CN, which is associated with decreased systemic inflammation and reversal of cellular immunity abnormalities [25,26]. These improvements may be especially advantageous in patients receiving immunotherapies but also play a role with VEGF-targeted therapies [27].

Finally, despite adjustments for established risk factors and the prognostic score, some unmeasurable variables may result in selection bias. These include the physician‘s subjective view of the patient’s general condition and comorbidities, as well as primary tumor properties, the extent and volume of metastatic disease, and surgical variables which are imperfectly represented by the prognostic models [28].

In our opinion supported by the results of the present analysis, patients with good performance status and one to two risk factors should be considered for CN. The case for CN is further strengthened after the inclusion of novel immunotherapies in mRCC treatment algorithms. In a phase II study analyzing survival and response rates in patients with mRCC treated with checkpoint inhibitors with/without CN, the treatment response rate and survival were clearly superior in patients after CN [29]. Few patients in the present cohort were defined as good prognosis, i.e., the interval from the diagnosis to the initiation of therapy was more than one year. These patients obviously had a more indolent disease that might have allowed for waiting on the decision to initiate therapy. However, a slight imbalance in the number of these patients in both cohorts probably had limited impact on the results that actually indicated a better outcome in the CN cohort that had only three of such patients.

Single-agent VEGF inhibitors are no longer the first-line treatment of choice for most patients with mRCC. Nevertheless, many issues identified in these studies, including delayed initiation of systemic therapy in exchange for tumor volume reduction, and delaying the emergence of treatment resistance by reducing tumor heterogeneity using CN, remain relevant in the immunotherapy era. 

The limitations of our study include the retrospective nature and associated potential bias. The population was not balanced according to the prognostic factors, similar to other retrospective studies, as demonstrated by Bex et al. [17]. Furthermore, inclusion of patients treated in higher lines with checkpoint inhibitors might have affected the survival results. Complete IMDC scoring could be calculated only in about 50% of patients. Therefore, full analysis of data based on IMDC was not possible. In addition, as pazopanib and sunitinib were not reimbursed by the public health insurance in the Czech Republic for the treatment of poor risk patients, few were included in the present analysis. 

## 4. Materials and Methods

### 4.1. Study Design and Data Source

The data were obtained from the RenIS Registry that represents a database of about 95% of all patients with mRCC treated with TT in the Czech Republic from 2007 to 2018 [30,31]. The database included a total of 4034 patients treated with targeted therapy for mRCC between 2006 and the end of January 2018. Patients with synchronous metastatic disease subsequently treated with first-line TT with pazopanib or sunitinib were included in the analysis. The CN/TT cohort included patients having CN within 3 months from the mRCC diagnosis and subsequently treated with TT, while the TT cohort included patients receiving TT upfront.

The RenIS registry contains information on the baseline patient characteristics, diagnosis, extent of the disease, baseline laboratory parameters, and type, outcome, and toxicity of treatments. The data were updated twice a year. Further details on the RenIS Registry were published elsewhere [20]. The project has been approved by the Multicenter Ethics Committee of the University Hospital and the Masaryk Memorial Cancer Institute in Brno, Czech Republic.

### 4.2. Statistical Analysis

Descriptive statistics and frequency tables were used to characterize the sample data set. The statistical significance of differences between subgroups was assessed using the Fisher exact test or the Mann–Whitney test.

Overall survival (OS) is defined as the time from the date of first treatment intervention to death due to any cause (i.e., for patients without nephrectomy, OS is computed from the date of the first line target therapy initiation, and for patients with nephrectomy OS is computed from the date of nephrectomy).

Progression-free survival (PFS) is defined as the time from the date of first treatment strategy (analogously to OS) to the date of the first documented progression or death due to any cause. 

PFS and OS were estimated using the Kaplan–Meier method, and all point estimates include 95% confidence intervals (95% CI). Statistical significance of differences in survival among subgroups was assessed using the log-rank test. 

Multivariable Cox proportional hazards models were used to evaluate the effect of all potential prognostic factors on the survival measures. Statistical significance of hazard ratios was assessed by mean of the Wald test. All statistical tests were performed at a significance level of α = 0.05. 

## 5. Conclusions

According to the results of this retrospective study, good and intermediate prognosis mRCC patients clearly benefited from CN. The benefit was particularly pronounced in patients with a single risk factor. CN was beneficial irrespective of tumor grade and age.

## Figures and Tables

**Table 1:** Baseline patient’s characteristics.

Characteristic	Cohort		*p*-Value ^(1)^
	CN/TT (*n* = 458)	TT (*n* = 272)	
**Sex**, *n* (%)			
Male	338 (73.8)	200 (73.5)	0.931
Female	120 (26.2)	72 (26.5)	
**Age at diagnosis** (years)			
median (range)	62 (25–83)	64 (35–85)	**0.006**
**Histology**, *n* (%)			
Clear cell carcinoma	431 (94.1)	261 (96.0)	0.581
Papillary cell carcinoma	22 (4.8)	8 (2.9)	
Chromophobe cell carcinoma	2 (0.4)	2 (0.7)	
Bellini duct carcinoma	2 (0.4)	0 (0.0)	
Unknown	1 (0.2)	1 (0.4)	
**Primary tumor grade**, *n* (%)			
G1–2 well/moderately differentiated	160 (34.9)	161 (59.2)	**<0.001**
G3–4 poorly/non differentiated	298 (65.1)	111 (40.8)	
**MSKCC score**			
good prognosis	3 (0.7)	7 (2.6)	**<0.001**
intermediate prognosis	417 (91.0)	215 (79.0)	
poor prognosis	38 (8.3)	50 (18.4)	
**MSKCC score—2 categories**			
good or intermediate prognosis	420 (91.7)	222 (81.6)	**<0.001**
poor prognosis	38 (8.3)	50 (18.4)	
**ECOG PS**			
PS < 2	433 (94.5)	258 (94.9)	0.999
PS ≥ 2	25 (5.5)	14 (5.1)	
**Calcium**			
≤2.5 mmol/L	422 (92.1)	231 (84.9)	**0.003**
>2.5 mmol/L	36 (7.9)	41 (15.1)	
**Hemoglobin**			
normal	273 (59.6)	147 (54.0)	0.163
<lower limit of normal	185 (40.4)	125 (46.0)	
**Time from diagnosis to target treatment**			
≥1 year	3 (0.7)	10 (3.7)	**0.006**
<1 year	455 (99.3)	262 (96.3)	
**LDH**			
≤1.5× upper limit of norm	428 (93.4)	229 (84.2)	**<0.001**
>1.5× upper limit of norm	30 (6.6)	43 (15.8)	
**Type of first target treatment**, *n* (%)			
sunitinib	361 (78.8)	210 (77.2)	0.643
pazopanib	97 (21.2)	62 (22.8)	
**Age at first target treatment initiation** (years)			
median (range)	63 (25–83)	64 (36–85)	**0.006**
**ECOG PS at first target treatment initiation**, *n* (%)			
PS 0	174 (38.0)	49 (18.0)	**<0.001**
PS 1	259 (56.6)	209 (76.8)	
PS 2	23 (5.0)	14 (5.1)	
PS 3	2 (0.4)	0 (0.0)	

CN/TT, cytoreductive nephrectomy followed by targeted therapy. TT, targeted therapy only; MSKCC, Memorial Sloan Kettering Cancer Center; ECOG, Eastern Cooperative Oncology Group; PS, performance status; LDH, Lactate dehydrogenase. ^(1)^ Fisher exact test or Mann–Whitney test. Bold: statistically significant.

**Table 2:** Progression-free and overall survival results: multivariable Cox-proportional hazards model.

Variable	Category	*n*	Progression-Free Survival		Overall Survival	
			HR	Wald Test *p*-Value	HR	Wald Test *p*-Value
			(95% CI)		(95% CI)	
**Nephrectomy**	**TT**	**271**	**1.000**	-	1.000	-
	CN/TT	457	0.629 (0.525–0.755)	**<0.001**	0.553 (0.449–0.681)	**<0.001**
**Sex**	female	191	1.000	-	1.000	-
	male	537	0.881 (0.730–1.063)	0.185	0.892 (0.717–1.109)	0.305
**Histology**	clear cell	692	1.000	-	1.000	-
	other histology types	36	1.961 (1.335–2.879)	**0.001**	1.810 (1.170–2.801)	**0.008**
**Primary tumor grade**	G1–2	319	1.000	-	1.000	-
	G3–4	409	1.300 (1.093–1.545)	**0.003**	1.453 (1.184–1.783)	**<0.001**
**MSKCC**	good or intermediate	640	1.000	-	1.000	-
	poor	88	1.359 (1.049–1.761)	**0.020**	1.931 (1.455–2.563)	**<0.001**
**Age at first target treatment initiation**	≤65 years	425	1.000	-	1.000	-
	>65 years	303	0.956 (0.806–1.135)	0.609	1.101 (0.903–1.344)	0.341
**First targeted treatment**	sunitinib	570	1.000	-	1.000	-
	pazopanib	158	0.788 (0.640–0.970)	**0.024**	0.825 (0.645–1.054)	0.123

CN/TT, cytoreductive nephrectomy followed by targeted therapy. TT, targeted therapy only; MSKCC, Memorial Sloan Kettering Cancer Center; HR, hazard ratio; CI, confidence interval. Bold: statistically significant.

**Table 3:** Progression-free survival and overall survival from first treatment strategy.

Number of Risk Factors (MSKCC)	Median PFS and OS	Cohort		Log-Rank Test *p*-Value
		CN/TT	TT	
MSKCC 1 risk factor patients, *n* = 328		*n* = 222	*n* = 106	
	Median PFS (95% CI)	10.2 months (7.8–12.5)	7.7 months (6.2–9.2)	**0.002**
	Median OS (95% CI)	37.9 months (28.1–47.7)	17.5 months (11.2–23.8)	**<0.001**
MSKCC 2 risk factors patients, *n* = 304		*n* = 195	*n* = 109	
	Median PFS (95% CI)	8.5 months (6.7–10.4)	5.8 months (4.7–6.8)	**<0.001**
	Median OS (95% CI)	21.9 months (16.2–27.5)	10.3 months (7.8–12.8)	**<0.001**

CN/TT, cytoreductive nephrectomy followed by targeted therapy. TT, targeted therapy only; MSKCC, Memorial Sloan Kettering Cancer Center; OS, overall survival; PFS, progression-free survival. The factors associated with adverse outcome in a multivariate analysis included the presence of liver metastases, grade 3 or 4 tumor, absence of CN, non-clear cell histology, and MSKCC poor prognosis (Table 2). Bold: statistically significant.

## Supplementary Materials

The following are available online at https://www.mdpi.com/2072-6694/12/10/2911/s1, Table S1: Metastatic sites at first targeted treatment initiation.
