# Supplementary material for: Cytoreductive Nephrectomy and Overall Survival of Patients with Metastatic Renal Cell Carcinoma Treated with Targeted Therapy—Data from the National Renis Registry
Source: Cancers (Basel). 2020 Oct 10;12(10):2911. doi: 10.3390/cancers12102911 (PMC7601448; doi:10.3390/cancers12102911)
Supplement: Supplementary file 1 [file cancers-12-02911-s001.pdf]

Supplementary Material: Cytoreductive Nephrectomy and Overall Survival of Patients with Metastatic Renal Cell Carcinoma Treated with Targeted Therapy – Data from the National Renis Registry

Alexandr Poprach, Milos Holanek, Renata Chloupkova, Radek Lakomy, Michal Stanik, Ondrej Fiala, Bohuslav Melichar, Katerina Kopeckova, Milada Zemanova, Igor Kiss, Igor Penka, Julia Bohosova and Tomas Buchler

Table S1. Metastatic sites at first targeted treatment initiation.

n (%)	Cohort		*p*-value[2)]
	CN/TT (n = 458)	TT (n = 272)	
**Site of Metastases[1)]**			
lungs	346 (75.5)	186 (68.4)	0.041
lymph nodes	178 (38.9)	160 (58.8)	**<0.001**
bones	155 (33.8)	97 (35.7)	0.809
liver	47 (10.3)	51 (18.8)	**0.003**
other visceral metastases	38 (8.3)	42 (15.4)	**0.007**
pleural effusion	14 (3.1)	20 (7.4)	0.017
peritoneum	12 (2.6)	17 (6.3)	**0.030**
brain	7 (1.5)	16 (5.9)	**0.003**
subcutaneous tissue	9 (2.0)	7 (2.6)	0.614
malignant ascites	4 (0.9)	7 (2.6)	0.115
skin	5 (1.1)	5 (1.8)	0.517
other localization	38 (8.3)	44 (16.2)	**0.002**
**Bones + liver metastases**			
bones negative, liver negative	273 (59.6)	143 (52.6)	**0.014**
bones positive, liver negative	138 (30.1)	78 (28.7)	
bones negative, liver positive	30 (6.6)	32 (11.8)	
bones positive, liver positive	17 (3.7)	19 (7.0)	

CN/TT, cytoreductive nephrectomy followed by targeted therapy. TT, targeted therapy only. [1)] One patient could have metastases in more sites. [2)] Fisher's exact test or Mann–Whitney test.
